# Garden cress seeds: a review on nutritional composition, therapeutic potential, and industrial utilization

**DOI:** 10.1002/fsn3.4096

**Published:** 2024-03-20

**Authors:** Tabussam Tufail, Tehreem Khan, Huma Bader Ul Ain, Sonia Morya, Mohd Asif Shah

**Affiliations:** ^1^ School of Food and Biological Engineering Jiangsu University Zhenjiang Jiangsu China; ^2^ University Institute of Diet and Nutritional Sciences The University of Lahore Lahore Pakistan; ^3^ Faculty of Health and Life Sciences INTI International University Nilai Malaysia; ^4^ Department of Food Technology & Nutrition Lovely Professional University Phagwara, Jalandhar Punjab India; ^5^ Department of Economics Kabridahar University Somali Ethiopia; ^6^ Centre of Research Impact and Outcome, Chitkara University Institute of Engineering and Technology Chitkara University Rajpura Punjab India; ^7^ Division of Research and Development Lovely Professional University Phagwara Punjab India

**Keywords:** functional foods, garden cress, good health, phytochemicals, therapeutics, wellbeing

## Abstract

The growing preference for natural remedies has resulted in increased use of medicinal plants. One of the most significant and varied plants is garden cress (*Lepidium sativum*), which has large concentrations of proteins, fatty acids, minerals, and vitamins. It also contains a wide range of bioactive components, including kaempferol glucuronide, gallic acid, protocatechuic acid, coumaric acid, caffeic acid, terpenes, glucosinolates, and many more. These substances, which include antioxidant, thermogenic, depurative, ophthalmic, antiscorbutic, antianemic, diuretic, tonic, laxative, galactogogue, aphrodisiac, rubefacient, and emmengogue qualities, add to the medicinal and functional potential of garden cress. An extensive summary of the phytochemical profile and biological activity of garden cress seeds is the main goal of this review. Research showed that garden cress is one of the world's most underutilized crops, even with its nutritional and functional profile. Consequently, the goal of this review is to highlight the chemical and nutritional makeup of *Lepidium sativum* while paying particular attention to its bioactive profile, various health claims, therapeutic benefits, and industrial applications.

## Graphical abstract

Garden cress is a highly nutritious plant that contains a significant amount of both macro‐ and micronutrients. Its seeds contain good quality protein and essential fatty acids, while the leaves comprise minerals such as calcium, iron, magnesium, and zinc. In addition to its nutritional value, garden cress is also an important medicinal plant, containing various phytochemicals with anticarcinogenic, antihypertensive, laxative, antidiabetic, and antioxidant properties. Research studies have confirmed that garden cress is safe for consumption without any side effects on the body.

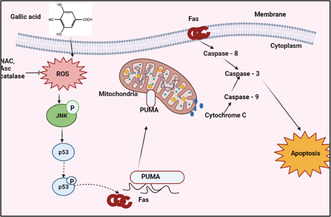

## 1 INTRODUCTION

Over time, there has been an increasing emphasis on the consumption of medical plants to combat various illnesses. This is because medicinal plants offer numerous benefits, including nutritional, bioactive, functional, and pharmacological advantages. Consequently, medicinal plants are becoming increasingly utilized in various food products on an industrial scale. Among the many medicinal plants (Mali et al., 2007), garden cress (*Lepidium stavium Linn*) is a widely used herb that belongs to the Cruciferae or Brassicaceae family. This fast‐growing plant reaches a height of 15–25 cm and is cultivated in various regions, including India, Asia, Europe, most African countries, and the United States, primarily as a salad plant. The garden cress features sessile leaves, small white flowers, and obovate or broad pods, all of which are edible. This annual herb can be grown throughout the year, although it flourishes best during the winter (Mali et al., 2007).

Garden cress seeds are distinguished by their tiny size, oval shape, and pointed triangular end. They are reddish‐brown in color and measure about 3–4 mm long by 1–2 mm wide. Polygonal cells with thick walls make up 80–85% of the seed, which is the endosperm (Jain & Grover, 2018). 2–3% of the seed is made up of the embryo, which is surrounded by endosperm cells, and 12–17% is made up of the seed coat. If the seed is submerged in water, it will quickly absorb the liquid. Additionally, the mucilage on the seed coat will expand and enclose the entire seed, producing a transparent and colorless covering (Jain & Grover, 2018).

Garden cress has captured greater importance in the field of food and nutrition because of the collaboration of different parts, including leaves, seeds, roots, and plants (Ramadan & Oraby, 2020). The utilization of all these parts has increased manyfold over time owing to the presence of different nutritional Likewise, the root is effective in tenesmus and secondary syphilis, and the plant is used to treat cough, bleeding piles, and asthma and as fodder for camels and horses (Ramadan & Oraby, 2020).

Even though fruit and vegetable juices are a wonderful source of vitamins and minerals, they lack protein and fat. Garden cress extract or powder may be used as a substitute for these components. While it is possible to make health‐promoting drinks by using both juice and extract, the combination of the two may result in beverages that are both tasty and nutritious. For moderate anemia, particularly in youngsters, the iron content in the seed powder might be an effective remedy (Eddouks & Maghrani, 2008). A group of scientists hypothesized that if the seeds are so medical, then the sprouts from the seeds should be even more medicinal, which led to the creation of large‐scale cultivation procedures for the plants in Europe (bin Abdullah Juma, 2007).

By keeping all the aforementioned perspectives in mind, it has been observed that garden cress is a highly neglible crop in spite of its important nutritional profile. Additionally, the literature demonstrated various efficacy studies regarding animals, but there is a dire need to conduct and increase efficacy trials on humans. Therefore, the current review has been designed to highlight the chemical and nutritional composition with special reference to the bioactive profile, different health claims, therapeutic effects, and industrial applications of *Lepidium sativum*.

## 2 NUTRITIONAL COMPOSITION

### 2.1 Chemical composition

Garden cress is considered a superfood due to its highly nutritious profile. It has a protein content of 21–25%, which is essential for muscle growth and tissue repair. Fat (23–25%) and carbohydrates (30–34%) make the seeds a good source of energy for those who are undernourished. Protein (24.2), fat (23.2), carbohydrate (30.7), fiber (11.9), ash (7.1), and moisture (2.9) were found in garden cress seeds (Zia‐Ul‐Haq et al., 2011). Seeds with low moisture content are more stable, more nutritious, and have a longer shelf life (Alsadee & Agbashee, 2021; Bathish et al., 2021). Various factors influence its proximate composition, such as plant species, seed variation, agronomic techniques, climatic and geographical conditions, and the stage at which seeds are gathered. Fruit and seed nutritional quality may be evaluated using this factor, and more research is encouraged on components that are more intriguing (Zia‐Ul‐Haq et al., 2011).

### 2.2 Vitamin and mineral profile

Garden cress is a rich source of vitamin C, also known as ascorbic acid, a water‐soluble vitamin originated from glucose metabolism (Valdés Fjad, 2006). It is a reducing agent required for the protection of body from free radical exposure and the synthesis of collagen fibers through proline and lysine hydroxylation. It is not synthesized in the human body due to the absence of glyconolactone oxidase. Garden cress is considered as a good source of ascorbic acid, and this property boosts its effectiveness against infection and diseases. Sat et al. (2013) reported that ascorbic acid content in *L. sativum* leaves ranges from 54 to 74 mg/100 g FW. Another study was also done to probe the ascorbic acid content in the stem, leaves, whole plant, and seeds of *L. sativum* and found 11.74 mg, 7.4 mg, 12.5 mg, and 9.68 mg, respectively (Malar et al., 2014). Moreover, thiamine (0.59 mg), riboflavin (0.61 mg), and niacin (14.3 mg) are present in appropriate amounts in this seed (Gokavi et al., 2004).

The major minerals present in garden cress are potassium and phosphorus, whereas the manganese content is low. It is also a good source of calcium, magnesium, and iron. Calcium (377 mg/100 g) and magnesium (430 mg/100 g) contents of garden cress aid in the correct contraction of muscle for healthy limb and heart motions. Anemia in youngsters may be treated with the seed's high iron content (100 mg/100 g). Phosphorus (723 mg/100 g) is essential for the body's normal metabolic functions. A study compared garden cress with different potassium sourced foods and found that garden cress has a higher potassium content than dates (696 mg/100 g), white beans (561 mg/100 g), spinach (558 mg/100 g), avocado (485 mg/100 g), and banana (358 mg/100 g) (Zia‐Ul‐Haq et al., 2012).

### 2.3 Fatty acids

Garden cress seeds contain 20–25% yellowish, semi‐drying oil. The fatty acid profile of garden cress seeds is characterized by high levels of linolenic (26–34%), linoleic (7.5–11.8%), and arachidic (2–3.5%), which are also helpful for the brain and memory. Palmitic, stearic, and oleic acids are also present in the seeds. Beta‐sitosterol and alpha‐tocopherol are found in the unsaponifiable materials. Garden cress oil contains 1422 parts per million (ppm) gamma and 356 parts per million (ppm) delta, 21 parts per million (ppm) alpha and a little amount of beta tocopherol (Ali, 2013). The fatty acid profile varies with the variation in seed variety, agronomic practices, and climatic and geographical conditions. In a study, found that fatty acid content varied among different cultivars of garden cress. In Khiderculivar, arachidonic acid was higher than linoleic acids, whereas in Haraz and Rajab cultivars, behinic and arachidonic acids were higher. Overall, it can be concluded that the nutrients in garden cress are a goldmine and that they may help to lower the incidence of many non‐communicable and nutritionally deficient diseases (Ali, 2013).

### 2.4 Amino acids

The seed protein of garden cress is of high quality and contains necessary amino acids such as lysine (6.26 g) and phenylalanine (5.65 g). Methionine (0.97 g) is a limiting amino acid in the seeds (Gopalan et al., 1971). Garden cress seeds contain all the essential amino acids, except for tryptophan and the S‐containing amino acids methionine and cysteine, which are only present in trace amounts. In addition, garden cress seeds are rich in two primary, non‐essential amino acids: glutamic acid and aspartic acid. The oilseed analysis showed that aspartic acid (9.76 ± 0.03%) and glutamic acid (19.33 ± 0.19%) were the most abundant amino acids in garden cress seeds (Abdel‐Aty, Bassuiny, et al., 2019; Abdel‐Aty, Salama, et al., 2019; Sedeeq et al., 2021).

The total essential amino acid percentage is 47.08%, with 6.26 ± 0.39% lysine and 0.97 ± 0.02% methionine. Tryptophan and cysteine were not determined. Essential amino acid score was 28.53% with methionine as the most limiting amino acid. Lysine helps to maintain proper nitrogen balance. The body uses methionine to derive the brain food, choline. It also aids in digestion, as well as serving as a fat burner. It can interact with other substances to detoxify harmful agents and is essential for the production of cysteine and taurine (Zia‐Ul‐Haq et al., 2012).

### 2.5 Phytochemicals

Garden cress *is* considered as a multifarious functional and medicinal plant as it possesses high levels of various phytochemicals such as alkaloids, terpenes, glucosinolates, and saturated and essential fatty acids in the seeds, leaves, seed oil, and roots. Some other important bioactive compounds in garden cress are gallic acid, protocatechuic acid, coumaric acid, caffeic acid, kaempferol glucuronide, and many others. The methanolic extract of defatted seeds has yielded phenolic components such as sinapic acid and sinapin (Oe & Gmelin, 1952). Flavonoids are the most prevalent and abundant class of plant polyphenols that can be obtained from a diet high in plants (Gupta & Morya, 2022). Subclasses of flavonoids (C6–C3–C6) may be divided into flavones, flavanones, flavanols, isoflavones, flavanols, chalcones, and anthocyanins, and they are usually found conjugated to sugars in the form of O‐glycosides or C‐glycosides, which are the most prevalent (Gupta & Morya, 2022). The seeds of *Lepidium sativum* have recently yielded two novel acylated kaempferol and quercetin chemicals, which have just been discovered. The total phenolic and flavonoid contents and antioxidant activity increased several times during the germination of garden cress seeds (Xiao et al., 2014).

High‐performance thin‐layer chromatography (HPTLC) was used to identify and quantify sinapic acid in a methanolic extract of Gc seed using the imidazole alkaloids semilepidinoside A and B. The sinapic acid was isolated from the glycosylates in Gc seed using a thin layer of silica gel and then analyzed using HPTLC‐photo densitometry. Glucotropaeolin 2‐phenyl ethyl glucosinolate, also known as gluconasturiin, was found, according to the researchers. Gc seed extracts and glucosinolates were also tested against the white fly, tabaci, for their insecticide properties. When pests were treated with glucotropaeolin, the death rate rose to 92.5% (Abdel‐Aty, Bassuiny, et al., 2019).

## 3 ANTI‐NUTRITIONAL COMPOSITION

Antinutritional compounds are those compounds that interfere with the absorption of nutrients in the body and metabolic processes and reduce the absorption of macro‐ and micronutrients (Singh et al., 2018). Garden cress seeds have a few anti‐nutritional compounds such as phytin phosphorus, oxalates, tannins, protease inhibitors, saponins, phytic acid, lectins, and amylase inhibitors. Among these compounds, phytin phosphorus and oxalates are mainly present in raw garden cress seeds (Azene et al., 2022).

## 4 BIOLOGICAL ACTIVITIES

Garden cress is a rich source of bioactive compounds, including gallic acid, protocatechuic acid, coumaric acid, caffeic acid, kaempferol glucuronide, and many others, which have been shown to have various health benefits, as mentioned in Table 1. These compounds exhibit anti‐inflammatory, anti‐carcinogenic, antihypertensive, laxative, and antioxidant properties (Morya et al., 2022). Additionally, garden cress seeds have a bitter taste and possess a range of therapeutic properties, including being thermogenic (promoting heat production), depurative (capable of purifying the body), ophthalmic (effective against eye diseases), antiscorbutic (prevent or cure scurvy), antianemic, diuretic (helps rid the body of salt and water), tonic (restoring and strengthening the nervous system), laxative (to treat constipation), galactagogue (induce, maintain, and increase milk production), aphrodisiac (arouses the sexual instinct, induces venereal desire, and increases pleasure and performance), rubefacient (produces redness of the skin), and emmenagogue (stimulates menstrual flow).

**TABLE 1:** List of bioactive compounds, structures, and functional properties of garden cress seeds.

Bioactive compounds	Structure	Amount (μg/100 g)	Biological activity
Gallic acid	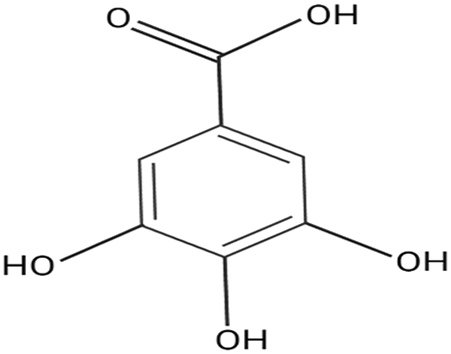	3001.75	Antioxidant, Antineoplastic, Anti‐inflammatory, Antihypertensive, Anti‐hypercholesteremia, Antiallergy activity
Coumaric acid	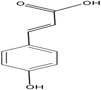	517.52	Antioxidant, Anti‐inflammatory, Antiapoptotic, Analgesic, Anti‐microbial activity
Caffeic acid	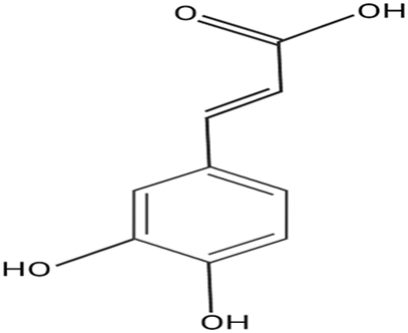	212.55	Antioxidant, Anti‐inflammatory, Anticarcinogenic, Anti‐mutagenic activity
Kaempferol	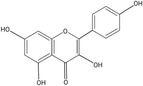	58.05	Anti‐inflammatory, Antioxidant, Hepatoprotective, Anticancer, Anti‐microbial activity
Quercitrin	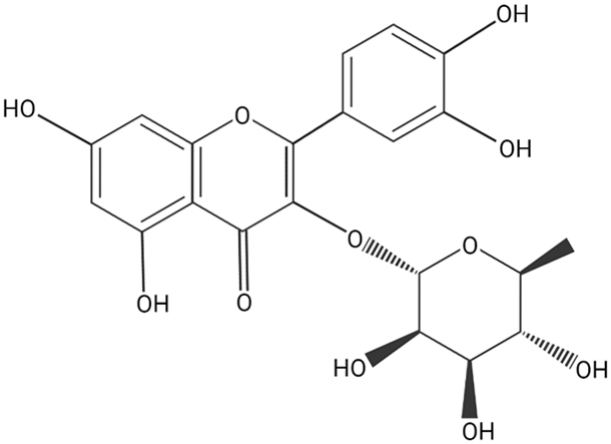	1520.33	Antioxidant and anti‐inflammatory effects might help reduce swelling, kill cancer cells, control blood sugar, and help prevent heart disease
Protocatechuic acid	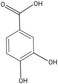	582.23	Antioxidant, Anti‐inflammatory, Anticancer, Anti‐asthma, Antibacterial, Antidiabetic, Antiulcer, Anti‐atherogenic activity

### 4.1 Antioxidant activity

The antioxidant activity of garden cress seeds is mainly due to the presence of phenolic compounds, phytosterols, and tocols, with tocopherols being the major contributor. Tocopherols act as scavengers of free radicals and inhibit oil oxidation, thus preventing various diseases. This property makes garden cress seeds highly valuable for the production of various dietary and functional products on an industrial scale. Moreover, garden cress seeds exhibit high DPPH antiradical activity, making them ideal for fortification in the development of various designer foods (Al‐Saad & Al‐Saadi, 2021).

Researchers looked into the antioxidant properties of the ethanolic extract of *Lepidium sativum* leaves, stem, whole plant, and seeds by different methods such as 1,1‐diphenyl‐2‐picrylhydrazyl (DPPH), total glutathione assay, reduced glutathione activity, and reducing power assay. The whole plant exhibited the greatest scavenging activity (12.1902%), while the stem exhibited the least amount (2.6905) (Malar et al., 2014).

Additionally, coumaric acid, another phenolic compound present in garden cress seeds, has been shown to possess antioxidant and anti‐inflammatory properties. Glutathione S‐transferase activity was highest in the seed (9600–56.3 g/mL) than in any other plant tissue studied. The ethanolic extracts of *Lepidium sativum* leaf had a lower glutathione concentration (9–0.2 g/mL). Alcoholic extracts were the most effective in the reducing power test (Kadam et al., 2018).

In a study, Kadam et al. (2018) explored the antioxidant activity of Garden cress ethanolic seed extract through different assays and found 187.12, 162.4, 119.32, and 35.29 μg/mL using superoxide scavenging activity, DPPH, metal‐chelating property, and ABTS, respectively (Attia et al., 2019).

### 4.2 Antidiabetic activity

The optimal dosage of 20% seed methanol extract of *L. sativum* to regulate blood glucose and treat hyperglycemia was 300 mg/kg BW. The methanol extract of *L. sativum* was able to regulate diabetes, increase antioxidants, and improve the lipid profile of the subjects (Kadam et al., 2018). In both NIDDM and healthy volunteers, the anti‐diabetic effects of *Lepidium sativum* seed powders were studied. Hypoglycemic activity was seen in a 21‐day therapy regimen.

More than 300 million people will have diabetes or chronic hyperglycemia by the year 2025. Free radicals are excessively produced because of the long‐term hyperglycemia. Compared to current synthetic medications, plant‐based herbal therapy is thought to be less harmful and devoid of side effects. If you've ever wondered what the name “habarachad” means, you're not alone. Saudi Arabia, Sudan, and other Arabic nations are known for their usage of this plant in the treatment of a variety of ailments. As a phytotherapy for hypertension, diabetic management, and renal illness or as a salad additive, *L. sativum* is often suggested (Eddouks et al., 2002).

### 4.3 Anti‐inflammatory effect

*Sativum* seeds contain 24 percent oil that is mostly composed of the omega‐3 and omega‐6 fatty acids, ALA and LA, respectively (12%). The antioxidants and phytosterols in this oil make it resistant to oxidation. The Wistar rats’ spleens and lungs showed synergistic effects of *L. sativum* oil (LSO) suppression of platelet aggregation and thromboxane B2 levels. Other studies have shown that LSO reduces lymphocyte proliferation and inflammatory mediator generation from peritoneal macrophages in rats (Diwakar et al., 2010).

Garden cress stems, leaves, and seeds are all good for your health (Jabeen et al., 2017). Tocopherol levels and antioxidant enzyme activity were raised in Wistar rats fed a diet enriched with LSO for 60 days (Jabeen et al., 2017). Phytochemicals present in garden cress seeds have shown antioxidant and anti‐inflammatory activity in various studies.

### 4.4 For the treatment of iron‐deficiency anemia

Research has shown that vitamic C supplementation helps improve iron absorption. It also boosts the digestive system's ability to absorb iron. Vitamin‐C and iron‐rich seeds of garden cress may be used to treat anemia or iron insufficiency without the need for additional supplements (Umesha & Naidu, 2015). In the stomach, L‐ascorbic acid helps to increase iron absorption by building a chelate with ferric iron at an acidic pH. This makes the iron more soluble at the alkaline pH of the duodenum, where it is more easily absorbed. Improves hemoglobin levels and cures anemia by consuming Garden Cress seeds on a long‐term basis. Garden cress seeds have a high concentration of iron, which encourages the development of red blood cells (Falana et al., 2014).

**FIGURE 1:**
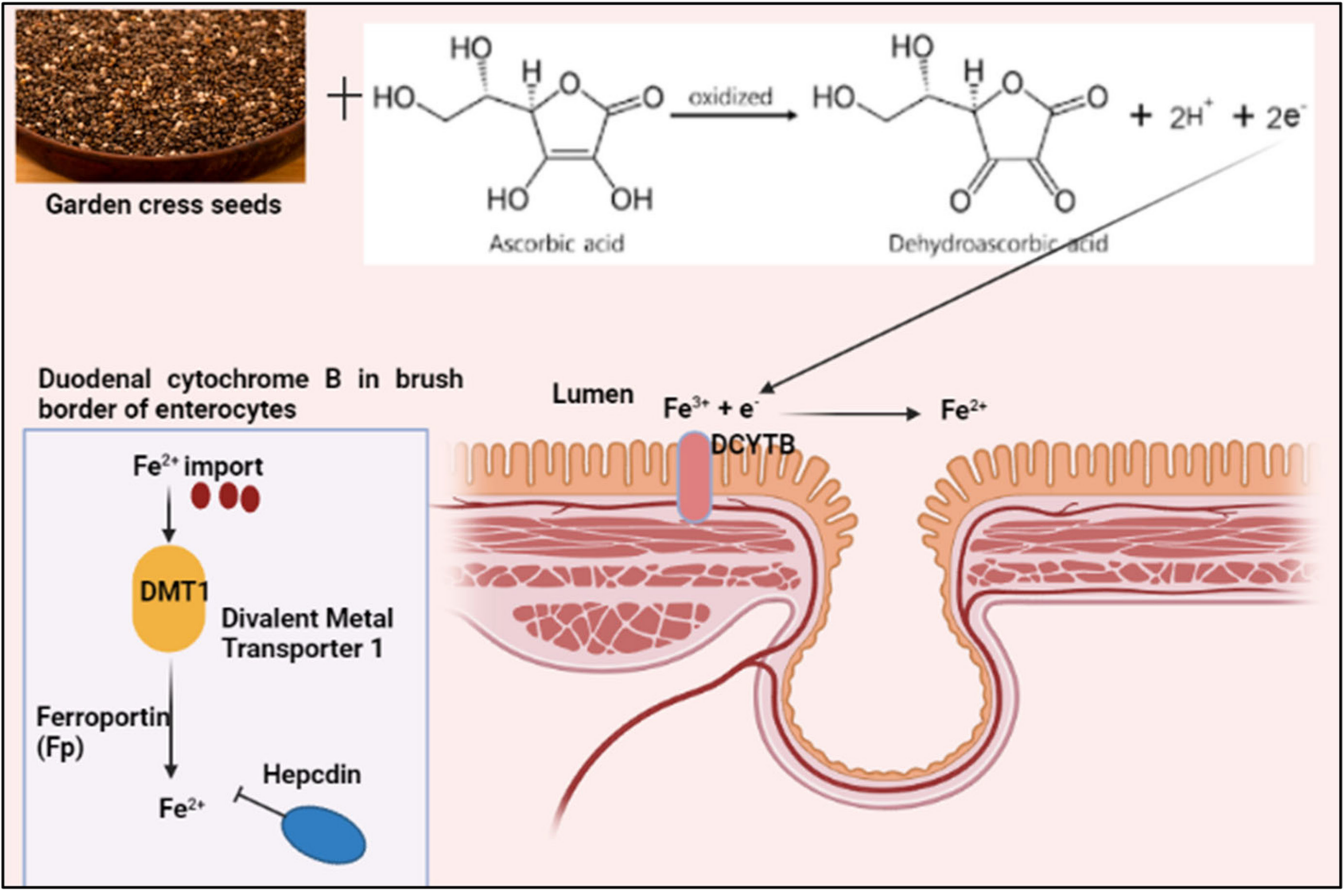
Mechanism of garden cress seeds in the treatment of iron‐deficiency anemia.

During adolescence, a person's physical and mental development accelerates. Adolescents with low iron levels may not be able to reach their full development potential because of a lack of iron in their diet. The seeds of garden cress are said to have galactogenic and antioxidant characteristics, as well as a great deal of promise as a functional food (Kumar et al., 2017), as mentioned in Figure 1.

### 4.5 Management of menstrual cycle disorders

Maintaining a regular menstrual cycle and having knowledge of the estimated date of conception are crucial for women throughout their lives. It is believed that the ingestion of garden cress seeds may assist in regulating menstrual cycles since the seeds contain estrogen‐like chemicals (Diwakar et al., 2010). On the subject of garden cress seed and reproduction, there is a wide range of opinions. It is also missing information on effectiveness, safety and how it works. It is also unknown how it affects LH secretion and reproductive function within the mammalian species studied. Rats (1.6 mg/g BW) given garden cress seed powder for 14 days showed a mammogenic and lactogenic effect. Aside from that, the ovariectomized rats administered a methanolic extract of LS for 21 days showed proceptive and receptive properties when 200 and 400 mg/kg BW were provided orally. A similar effect on prolactin, progesterone, and luteinizing hormone in ovariectomized rats has been observed (Patel et al., 2009).

### 4.6 For breast milk secretion

Nutritional significance for breastfeeding mothers is well known with the seeds of garden cress. It aids the nursing mother in sustaining the flow and production of breast milk that is rich in protein and iron (Imade, Erinfolami, et al., 2018; Imade, Smith, & Gazal, 2018). Garden cress seeds have several properties, including being a tonic, demulcent, rubefacient, carminative, galactagogue, and emmenagogue. It has been found to help an average of 11 nursing mothers produce more milk throughout the postnatal period. Additionally, it is often recommended for those suffering from diarrhea or dysentery (Prajapati & Dave, 2018). Syphilis and Tenesmus may be treated with the root, which is bitter, caustic, and used as a condiment. Male aphrodisiac effects and an increase in testosterone concentration have been linked to garden cress, whereas female anovulatory and abortifacient effects have been linked to garden cress (Uphof, 1959).

Breast milk is an excellent source of nutrition and immune system support for infants. Graden cress seeds are excellent galactagogues, and their consumption is highly recommended for nursing mothers (Khan, 2018).

### 4.7 Anticancer activity

**FIGURE 2:**
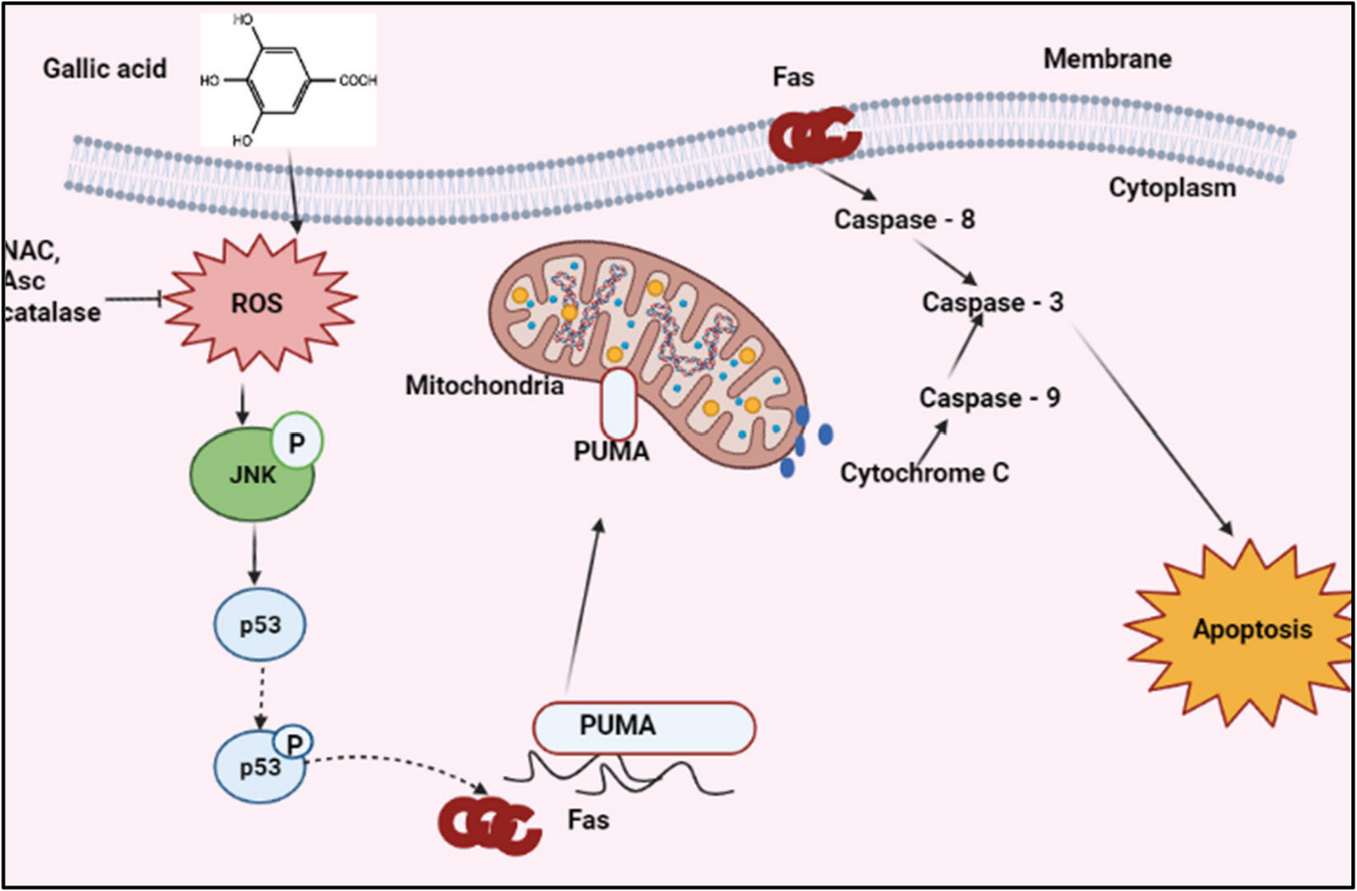
Mechanism of gallic acid's anticancer activity.

Recent research suggests that garden cress may be used to combat cancer. This product includes antioxidants, including Vitamin A and Vitamin E, which protect cells from free radical damage. Additionally, it contains a bioactive compound that has been shown to inhibit the synthesis of enzymes that can lead to tumor growth. Studies have demonstrated that the injection of this compound is effective in killing breast cancer cells (Imade, Smith, & Gazal, 2018). Antioxidant, anti‐inflammatory, and cancer‐protective properties have been found in *L. sativum*'s phytoesterols as well as their metabolites (Singh et al., 2015). Phenolic chemicals, particularly flavonoids, may protect the human body against oxidative stress, which may contribute to cancer, aging, and cardiovascular illnesses. For example, Table 1 indicates that gallic acid has been found to inhibit apoptosis and cancer‐causing pathways in the body (Conforti et al., 2008; Hazafa et al., 2022; Singh et al., 2015) (Figure 2).

Alternative and natural breast cancer treatments are being investigated to minimize or eliminate the drawbacks of traditional therapies. Many chemo‐preventive and therapeutic components have been identified in the seeds of *L. sativum* L. (garden cress) (Hazafa et al., 2020). Flavonoids found in *L. sativum* seeds and leaves were tested for their cytotoxic effects on Hep. G2 cells. *L. sativum* seed O‐glycoside extract (ethyl acetate extract) exhibited the highest cytotoxic impact on Hep. G2 cells, with butanol seed extract in third place. Kaemferol present in the garden cress seeds has been found to inhibit many forms of cancer, such as lung cancer, brest cancer, brain tumors, prostate cancer, and hepatic cancer (Mahassni & Al‐Reemi, 2013).

### 4.8 Healing properties of *Lepidium sativum* for bone fractures

There are several health benefits associated with the seeds of *Lepidium sativum*, including the healing of fractures, and variables that might delay or hinder this healing process. The plant's anatomical parts, especially the seeds, exert their effects. Some research was performed on animal models to assess the healing properties of graden cress, and it was noticed that garden cress increased collagen deposition and tensile strength at the fracture sites and radiologically accelerated callus development in rabbits (Ahmad et al., 2021; Bin Abdullah Juma & Martin, 2011).

The mucilage extracted from the dried seed coat of garden cress seeds has been used as an excipient in various pharmaceutical formulations (Dixit Jr et al., 2020). The use of *L. sativum* caused a noticeable increase in collagen deposition at the location of the fracture, which was connected to a considerable improvement in the fractured tibia's tensile strength. This investigation supports the plant's traditional use as a bone‐healing agent (Prajapati et al., 2014).

In another study, the test group's diet included *Lepidium sativum* seeds, and x‐rays were utilized to track the healing status of the fracture over the course of 6–12 weeks. Anecdotal evidence and statistical evidence suggest that the traditional usage of *Lepidium sativum* for speeding up bone fracture healing is justified. Studies like this demonstrate the necessity for more investigation into this topic (Ahsan et al., 1989).

In Saudi Arabia and other Arabic nations, plants and seeds are widely accepted as useful mediators for the healing of damaged bones. Research was done on six adult New Zealand white rabbits to demonstrate this effect. Everyone had fractures in the middle of their left femur, which were classified into two categories. During the experiment, one group of rabbits had regular food mixed with garden cress seed, whereas the other group received simply a normal diet (Ahmad et al., 2021). X‐rays of the induced fractures were collected at 6 and 12 weeks postoperatively to monitor the healing process. Test group fracture recovery was statistically superior to the control group, according to the findings (Ahmad et al., 2015).

### 4.9 Osteoarthritis intervention activity

Research was conducted to investigate the efficacy of *Lepidium sativum* seeds in treating osteoarthritis. The study involved two groups of patients. A control group received starch as a placebo, while the drug group received *Lepidium sativum* powder split into two daily doses of 6 gm each for a period of 30 days. Evaluations of osteoarthritis symptoms were conducted before and after treatment to determine whether there were any improvements. Results showed that more than three‐quarters of patients in the drug group experienced significant improvement in symptoms, with 30% achieving full remission, 37.5% showing substantial progress, and 25.5% reporting moderate progress (Ahmad et al., 2015; Al‐Snafi, 2019). The seeds have been shown to accelerate glucocorticoid‐induced osteoporosis in rats. Conversely, they have been found to promote faster healing of artificial fractures in the midshaft of the left femur in rabbits. However, the anti‐osteoporotic effects of the seeds and the underlying mechanisms have not been studied in ovariectomized rats (bin Abdullah Juma, 2007; Rahman et al., 2004).

It has been suggested that *Lepidium sativum* can be a useful addition to current osteoarthritis treatment regimens due to its analgesic and anti‐inflammatory properties, as well as its high concentration of Ca^2+^ ions. Only a small number of patients in the control group showed improvement, which can mostly be attributed to the eradication of causal factors that was provided to all patients in both groups (Rahman et al., 2004).

### 4.10 Anti‐hypercholesterolemic effect

A study demonstrated that feeding rats with a diet supplemented with garden cress seed oil led to significant reductions in triglycerides, total cholesterol, LDL‐c, VLDL‐c, and AST and ALT enzyme levels compared to the positive control group. Interestingly, HDL levels were found to be slightly elevated in rats given varying doses of garden cress oil. These findings suggest that a diet rich in garden cress oil could have beneficial effects on the blood lipid profile and plasma lipoproteins in individuals with dyslipidemia (Kraft, 2015). Garden cress seeds have not yet been studied for their cholesterol‐raising properties. Unripe carnauba fruit aqueous pulp extracts were further studied for their hypocholesterolemic efficacy in experimental animals fed a high‐cholesterol diet (Youssef et al., 2014).

### 4.11 Effect on bronchial asthma/digestive functions

Sore throats, asthma, headaches, and coughing may be alleviated with the use of garden cress seeds. Due to the fact that it has the properties of a bronchodilator, it is recommended for patients with bronchitis (Imade, Erinfolami, et al., 2018; Khan, 2018). A study was conducted on 30 male and female patients aged 15–80 with mild to moderate bronchial asthma, excluding pregnant women. The patients were administered 1 g of finely powdered seed powder orally, three times a day, for a period of 4 weeks. The spirometer results indicated significant improvements in several pulmonary functions during and after the study period, with no reported adverse effects among the subjects (Doke & Guha, 2014).

Table 2 indicates that the ingestion of a large quantity of goiter can lead to hypothyroidism. However, *Lepidium sativum* L. has demonstrated its effectiveness in treating bronchial asthma, hiccups, coughs with expectoration, and bleeding piles. Several Ayurvedic practitioners have recommended the use of *L. sativum* seeds (Rehman et al., 2012). There are a variety of medical uses for the plant. Patients with bronchial asthma benefit from the diuretic and mildly stimulating properties of the leaves of this plant. *L. sativum*, on the other hand, has not been studied in terms of its ability to cure bronchial asthma (Chopra et al., 1986).

**TABLE 2:** Systematic pharmaceutical case studies and key findings of garden cress.

Title of the study	Study information	Key findings
Pharmacological and safety evaluation studies on *Lepidium sativum* L., seeds	The seeds are considered galactagogue, emmenagogue, depurative, and aphrodisiac. Seeds possess significant anti‐inflammatory, coagulant activities, and antipyretic	Can cure scurvy, dyspepsia, diarrhea, leucorrhoea, and seminal weakness
Importance of n − 3 fatty acids in health and disease	Normal rats exhibited a substantial increase in glomerular filtration rate following oral administration of LS	Found to affect diabetes and hypertension
Study of the hypoglycaemic activity of Lepidium sativum L. aqueous extract in normal and diabetic rats	The hypoglycaemic activity of garden cress seed in aqueous extract in diabetic and normal rats	It showed that the plasma insulin in diabetic became normal when treated with an aqueous extract of garden cress seed
Local use of spices, condiments, and non‐edible oil crops in some selected woredas in Tigray, Northern Ethiopia	In Ethiopia, the garden cress seed powder is mixed with water, lemon, garlic, salt, and pieces of injera to increase its therapeutic value	Treatment to prevent infection Stomach ache, toothache, and abdominal pain
Antidiarrheal activity of methanolic extracts of seeds of Lepidium sativum	Studied the antidiarrheal activity of methanol‐extracted garden cress seed in castor oil‐induced diarrhea in mice	It showed high antidiarrheal activity since it affects fluid secretion and gastrointestinal propulsion
Conventional nutrients and antioxidants in garden cress seeds (*Lepidium sativum*): an explorative and product development endeavor	Garden cress seed in both forms, whole garden cress seed powder and roasted garden cress seed, is high in phenols and nutrients, and the antioxidants present aid in the treatment of many health problems	A Plethora of diseases can be treated
“In vitro” Antimicrobial Assessment of “*Lepidium sativum*” L. Seeds Extracts	Studied the antimicrobial activity of garden cress seed extract with different solvents like water, methanol, and petroleum ether against six different varieties of microbes like fungus *Candida albican*, *Pseudomonas aeruginosa*, *Staphylococcus aureus*, *Klebsiella pneumonae*, *Proteus vulgaris*, and *Escherichia coli*	It was found that petroleum ether extract has great antimicrobial activity against the six microbes
Fracture healing activity of ethanolic extract of *Lepidium sativum* L. seeds in internally fixed rats' femoral osteotomy model	Studied the effect of ethanolic extract of garden cress seed on fracture healing ability by femoral osteotomy model and by taking x‐rays after a specific time gap in mice and compared it with the control group, which was not given the extract	It showed that the garden cress seed ethanolic extract has a great effect on fracture healing
Evaluation of the antifungal effect on ethanolic extract of *Lepidium sativum* L. seeds	Studied the antifungal activity of an ethanolic extract of garden cress seed, especially against *Alternaria alternate*, *Fusarium equiseta*, and *Aspergillus flavus* Potato Dextrose Agar (PDA)	It proved that the ethanolic extract of garden cress seed has high antifungal activity
Lepidium sativum Linn.: A current addition to the family of mucilage and its applications	The dried leaves are used to cure different diseases	Used as a diuretic to alleviate inflammation and muscle pain, rheumatism, and bronchitis
A review article *Lepidium sativum* (garden cress)	When the garden seed is mixed with lime juice, it increases the health benefits	Reduce inflammation and rheumatic pain
Formulation and sensory evaluation of food products developed by incorporating germinated garden cress seeds (*Lepidium sativum* L.)	We can use germinated garden cress seeds in different cooking methods and increase the nutritive value of foods	It can be used to treat poultices for sprains, asthma, skin disease, coughs, and dysentery diarrhea
Enhancing nutritional quality of corn extruded snack by incorporating moth bean (vignaaconitifolia) and garden cress seeds (*Lepidium sativum*)	Along with this, molasses and coconut kernel garden cress seed powder have numerous health benefits	Can reduce anemia
Improved shelf‐life and consumer acceptance of fresh‐cut and fried potato strips by an edible coating of garden cress seed mucilage	A mucilage coating made from garden cress seed is added to the potato after cutting and studied for shelf life and oil intake while frying	It is observed that the browning reaction and the shelf life of the potato are improved after the addition of the garden cress seed mucilage and also show little intake of oil after frying
Reproductive performance and milk yield of rabbits fed diets supplemented with garden cress (*Lepidium sativum*) seed	The reproductive and milk‐yielding capacities of rabbits are studied while giving the garden cress seed as a supplement	It showed that the garden cress seed has a positive impact on the reproductive as well as milk‐yielding of rabbits
Garden Cress (*Lepidium sativum*) seeds ameliorated aluminum‐induced Alzheimer's disease in rats through antioxidant, anti‐inflammatory, and antiapoptotic effects	The study examined the impact of the administration of garden cress seed extract on biochemical, cerebral, histopathological, and behavioral, and changes in rats with AlCl_3_‐induced AD and explored the mechanism behind this effect	It showed changes due to its antioxidant, antiapoptotic, and anti‐inflammatory effects, suggesting that it has a neuroprotective effect

### 4.12 Antihypertensive effect

The objective of this section was to study the antihypertensive properties of *Lepidium sativum* L. on spontaneously hypertensive rats (SHR). *Lepidium sativum* L., a plant belonging to the *Lepidium sativum* family, has been found to have hypotensive and diuretic effects on both normotensive (WKY) and spontaneously hypertensive rats (SHR) (Paranjape & Mehta, 2006). Aqueous LS extract (20 mg/kg for 3 weeks) significantly reduced blood pressure in SHR rats, while no significant reduction was seen in WKY rats. Treatment with SHR rats considerably reduced the systolic blood pressure between day 7 and day 30. However, in WKY rats, LS extract considerably increased water excretion (*p* = .001), while in SHR rats, there was no statistically significant difference (Wadhwa et al., 2012).

As a result, WKY rats were given 20 mg/kg aqueous LS extract orally, and the excretion of sodium, potassium, and chlorides in the urine was significantly increased. The aqueous LS extract treatment increased sodium, potassium, and chloride urine elimination in spontaneously hypertensive rats (Kagathara et al., 2009). Table 2 indicates that normal rats exhibited a substantial increase in glomerular filtration rate following oral administration of LS, but SHR rats showed no significant change throughout the treatment period. Furthermore, there were no significant changes in the heart rate of SHR and WKY rats following LS therapy (Maghrani et al., 2005; Verma et al., 2021).

## 5 INDUSTRIAL APPLICATIONS

### 5.1 Food industry

Over a period of 6 months, a group of researchers investigated the effects of a health mix that included garden cress seeds on a sample of 100 teenage females who were suffering from anemia in the Dindigul area of Tamil Nadu (Besufekad et al., 2018). The health mix, which contained 5 g of garden cress seeds, 20 g of rice flakes, 5 g of bajra, 5 g of roasted Bengal gram dhal, and 5 g of samai, provided 10 mg of iron in a 50‐g serving. The researchers monitored the participants' hemoglobin levels before and after the six‐month supplement period. The results showed a significant increase in mean hemoglobin levels from 8.23 ± 0.49 to 11.11 ± 0.46 after the follow‐up period (Azimkhanova et al., 2021).

A second study investigated the impact of garden cress ladoo on the hemoglobin levels of anemic 12‐ to 15‐year‐old females. The study included 100 moderately undernourished girls, who were divided into two groups: an experimental group and a control group. The control group did not receive any deworming or supplementation. After 6 months, the experimental group had a significant increase of 3.8 g/dL in hemoglobin levels, while the control group showed a slight rise of only 0.1 g/dL. The experimental group also reported a decrease in anemic symptoms such as exhaustion and overall weakness, whereas the control group showed no significant changes (Abuelgasim et al., 2015).

**FIGURE 3:**
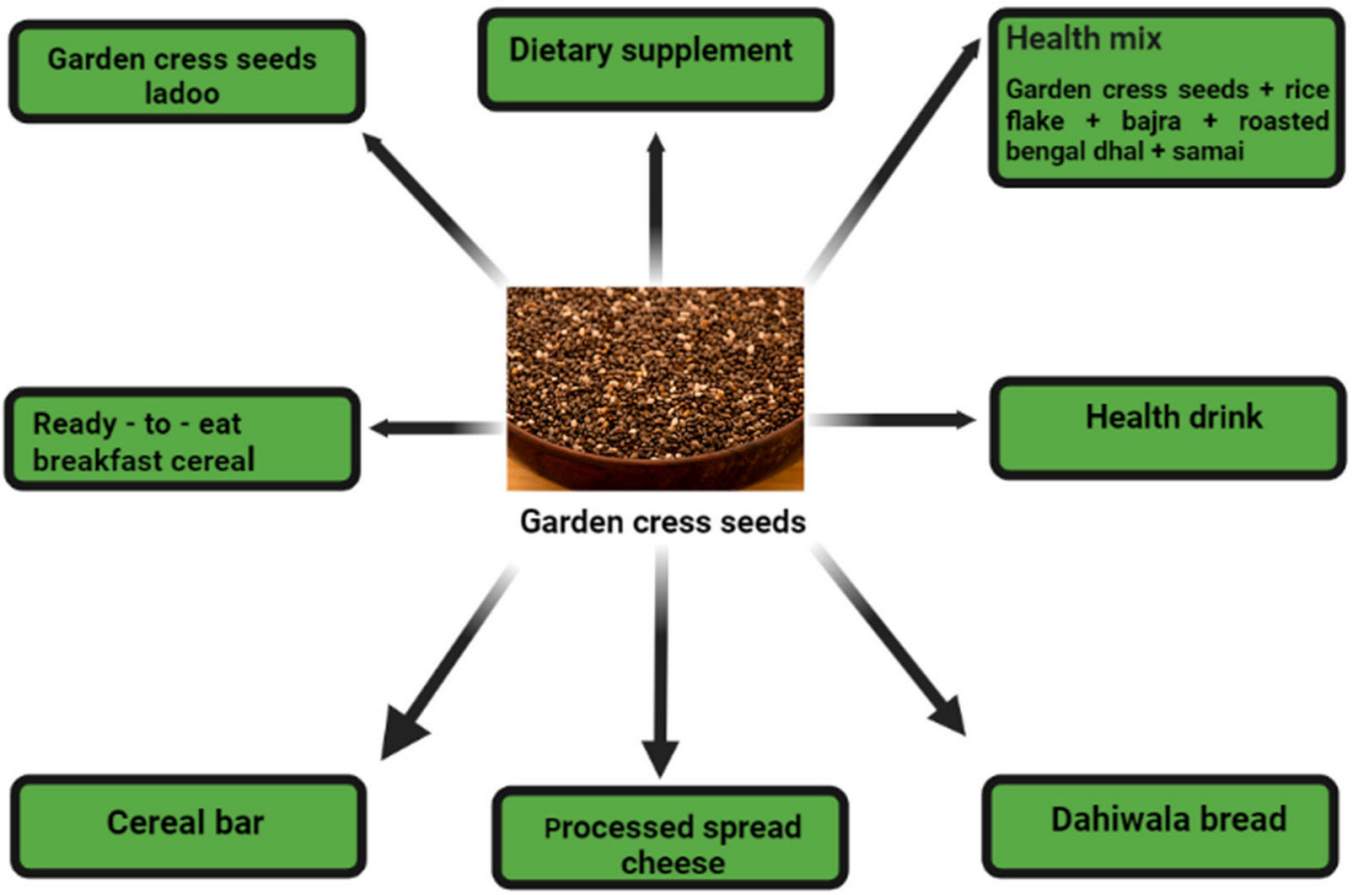
Food products of garden cress seed.

When it comes to cakes and breads of all kinds (including a variety of multigrain varieties), they've recently found their way into salad dressings and soup mixes as well as in ready‐to‐eat morning cereals and beverages like cereal bars (Abo El‐Maati et al., 2016) (Figure 3). Some food items and bakery goods use flaxseed as a nutritious component, and its flour is used in the production of bread. Ethiopians eat the seeds, which are boiled, crushed, mixed with seasonings and water, served with Injera and breads, used to prepare food and edible oil, and also consumed as a porridge and beverage called chillika (Angel & Devi, 2014).

### 5.2 Dahiwala bread

Dahiwala bread, a simple breakfast meal, is made by combining bread slices with curd and baking them. To reduce the amount of saturated fat in the bread, the mixture is cooked in soybean oil. Additionally, garden cress seeds are added to boost the nutritional value of the bread, increasing its protein, fat, calcium, iron, and phosphorus content. Due to its medicinal characteristics, this product can be both nutritious and beneficial for treatment purposes (Angel & Devi, 2015).

A recent study has shown that the addition of Garden Cress Seeds (GCS) can improve the rheological qualities and overall quality of rice–wheat bread (Bhardwaj et al., 2020). Four different amounts of G and L hydrocolloids (0%, 0.3%, 0.6%, and 1%) were added to the flour to create composite bread. The study found that GCS served as a novel and effective gluten substitute, enhancing the quality and texture of the bread. These properties could be particularly beneficial for functional food preparations aimed at addressing issues such as milk production and anemia in women (Eyres & Eyres, 2014).

### 5.3 Health drinks

Garden cress seeds are a rich source of protein, carbohydrates, and critical minerals such as calcium, iron, and phosphorus, as well as crude dietary fiber (7.6%). These seeds are commonly used to make health beverages with milk, such as milk‐based health drinks. Typically, these drinks contain 5% sugar (w/v), 1% fat, and 3% processed garden cress seeds (Agarwal & Sharma, 2013). This product is beneficial for a wide range of individuals, including children, the elderly, newborns, and convalescent patients. The milk‐based beverage enriched with Garden Cress seeds contains all the vital nutrients required for these individuals, such as protein, carbohydrates, calcium, iron, phosphorus, and crude dietary fiber. Consuming this supplement can help prevent nutritional deficiencies and promote lean muscle growth, especially in those who regularly engage in physical activity.

In a recent study, researchers examined the effects of Garden Cress seed oil on lipid metabolism by combining it with vegetable oil. Garden cress oil is high in α‐linolenic acid, an essential omega‐3 fatty acid. Traditionally, vegetable oils such as sunflower, maize, safflower, and soybean oil have been recommended to have a 10:1 or 2:1 ratio of n‐6 to n‐3 PUFA. However, the study found that blending Garden Cress seed oil with other vegetable oils could produce a PUFA ratio of 2.3–2.6. Rice bran oil, sunflower oil, and sesame oil were used in the experiments (Zanvar & Rohini, 2007).

### 5.4 Uses of animal feed additives in broilers

Additives for animal feed are utilized all over the globe for a variety of reasons. To ensure that animals get the necessary nutrients they need, certain feed additives are included in their diets. Other feed additives are added to increase animal performance by increasing the amount of food they consume and their ability to develop (Salehi, 2019; Schmitz & Ecker, 2008). Anti‐microbial drugs in animal feed are not permitted in many countries throughout the globe because of the current trend in animal nutrition toward more “natural” diets (Umesha & Naidu, 2012). This has sparked a renewed interest in the feed sector to explore alternatives that may be acceptable to the public. Recent studies have shown that probiotics, prebiotics, herbs, spices or botanicals (e.g., essential oils), enzymes, and minerals are all excellent alternatives. The use of herbs, spices, and their extracts can have a significant impact on the health of chickens in multiple ways. All of these properties contribute to the development of immunity and growth by providing antioxidant and antibacterial properties. Regulating feeding and stimulating digestive secretions may improve digesting capacity and lower the risk of digestive problems (Zeilab Sendijani et al., 2020).

Herbs have been used for medicinal purposes since ancient times due to their availability, low side effects, and effectiveness in treating various ailments in both humans and animals. However, studies on the use of herbal combinations in broiler diets have yielded mixed results. While some researchers have reported improved broiler performance, others have found no significant impact on body weight gain, feed intake, or feed conversion. In Ethiopia, *Lepidium sativum* has been traditionally used by farmers for both medicinal and dietary purposes, highlighting its potential as a valuable ingredient in animal feed (Strasser et al., 2000).

The leaves, roots, and seeds of garden cress have a long history of traditional use in many countries as a bone fracture healing agent, anti‐asthmatic, antiscorbutic, aperient, diuretic, galactogogue, poultice, stimulant, and blood pressure regulator. Garden cress seeds are also known to have a positive impact on weight gain due to their high concentration of n‐3 polyunsaturated fatty acids, tocopherols, lignans, and antioxidants (Taghipour et al., 2021).

### 5.5 Dietary supplements

Studies have found that the cress seed had a high (86.90%) water absorption capacity (229 mL H_2_O/100 g). Garden cress seed can be used as an ingredient in the meat, bread, and cake industries. Aside from being a nutritious supplement or nutrient substitute, the protein isolated from the seed of garden cress can be used in food systems (Shawle et al., 2016). Cress seed is rich in iron, protein, and calcium, and it may be used in the treatment of various health problems. A wheat‐based food product like Mathri can be prepared by using cress seed powder at different levels (2.5%, 5%, and 7.5%). Particularly, 5% husk‐removal GCSP was found to have a higher nutritional value than standard mathri, as well as the ability to protect against oxidative stress (Zia‐Ul‐Haq et al., 2012).

### 5.6 Role in the processed spread cheese

Cheese‐based foods, such as sandwiches, hamburgers, pizza, and lasagna, often use processed cheese products as an ingredient. Sadly, certain emulsifiers used in the manufacturing of cheese have been linked to health issues (Hacıseferoğulları et al., 2005). Sodium phosphate, a common component in processed cheese products, has been shown to cause kidney damage. Additionally, tartrate may produce diarrhea, and potassium phosphate might lead to an allergic response. As a result, there is still a pressing need to find novel emulsifying agents (Ghamari et al., 2021).

In a study, cress seeds were used to make processed spread cheese rather than salt as an emulsifying agent. When making processed spread cheese, cress seed powder was made and applied in six different ratios (0.05%, 1.5%, 2.5%, 3.5%, 4.5%, and 5.5%) in contrast to the control (3% commercial emulsifying salt). Results showed that 3.5% cress seeds showed significant health‐enhancing qualities, particularly in decreasing glucose ratios (Punia & Dhull, 2019).

*Lepidium sativum* is a good source of protein and dietary fiber, as well as vital amino acids. Cress seeds have been shown to be safe and non‐toxic by toxicology investigations. Cress seed has been shown to be beneficial in the treatment of hypertension, renal illness, and diabetes. Hypocholesterolemic and antidiarrheal qualities are among its numerous therapeutic benefits. Fracture healing, diuretic, and nephroprotective properties are also present. Galactogogue and anti‐inflammatory and antipyretic properties are also present (Mohamed & Hussein, 2017).

### 5.7 Natural super‐disintegrant

Disintegrants are commonly used in tablet formulations to facilitate faster dissolution by breaking them down into smaller particles. Various studies have been conducted to compare the effectiveness of natural and synthetic superdisintegrants. In one such study, mucilage obtained from lentil seeds (*L. sativum*) was used to develop fast‐dissolving tablets of Nimesulide, a nonsteroidal anti‐inflammatory drug (NSAID) (Ramadan & Oraby, 2020). The study revealed that using a 10% mucilage concentration resulted in a greater dissolution of the tablet. Additionally, mucilage derived from lentil seeds was used to create oral disintegrating tablets of Metformin HCl through the direct compression process (Saju & Sivaraman, 2021).

Garden cress seeds contains mucilage, which functions primarily based on swelling mechanisms that act as binders and disintegrating agents. Compared to leaves, seeds often have a higher mucilage concentration (Iswariya et al., 2021). In fact, research comparing natural and synthetic superdisintegrants found that *L. sativum* mucilage performed better overall than the synthetic alternative (Dixit Jr et al., 2020).

### 5.8 Controlled‐release system application

Solid controlled‐release oral unit dose pharmaceutical compositions have been developed for oral administration using hull powder from *L. sativum* seeds. The compositions contain one or more medicines and the gel‐forming husk powder from *L. sativum* seeds in concentrations ranging from 10% to 70% of the total weight of the dose form. A novel interpenetrating polymer network (IPN) composed of *L. sativum* and PVA was constructed for the first time by the researchers, who then employed the IPN as a cross‐linker to generate microspheres containing simvastatin as the model medication by cross‐linking the microspheres utilizing emulsion cross‐linking (Rani & Ahuja, 2017).

## 6 OTHER APPLICATIONS

Garden cress gum and maltodextrin were used as microencapsulation coats for the entrapment of garden cress phenolic‐rich extract for improving its storage stability and antioxidant and antibacterial activities (Abdel‐Aty et al., 2023). Cress seed mucilage, either alone or in combination with a synthetic polymer, may be utilized to provide a novel drug delivery mechanism for patients (Moreira et al., 2019; Nerkar & Gattani, 2012). Cress seed gum improved the dough's extensibility value from 0.3% to 0.6%, followed by a decrease at 1%. As cress seed gum concentrations rise, bread's water activity and crumb firmness both deteriorate. This has led to the development of novel edible films and coatings based on cress seed gum. In addition to its inexpensive cost of manufacture and hydrophilic, biocompatible, and biodegradable characteristics, it also has strong rheological qualities (Razmkhah et al., 2016).

Since the Vedic period, it has been regarded as a major source of nourishment and medicine because of its health‐enhancing characteristics. Garden cress aids in the removal of toxins from the body and increases food intake. Functional meals may be made from the entire edible seed (Uphof, 1959).

## 7 CONCLUSIONS

The present review thoroughly examined the nutritional makeup, potential for therapeutic use, and industrial application of garden cress seeds. The nutritional profile highlights the seeds' abundance of important nutrients, antioxidants, and bioactive substances, establishing them as a desirable dietary supplement. Garden cress seeds have proven to have anti‐inflammatory, antioxidant, anti‐cancer, and antibacterial qualities, according to research on their therapeutic potential. Literature has demonstrated that garden cress seeds have a positively significant effect on the growth of children and adolescent girls. The seeds' potential as functional food ingredients and nutraceuticals is suggested by their ability to positively impact health disorders like diabetes and cardiovascular illnesses. The analysis also examined garden cress seeds' industrial uses, highlighting their adaptability in the food, pharmaceutical, cosmetic, and agricultural industries, further emphasizing their potential role in sustainable industrial practices. It is clear from this review that garden cress seeds are an important but little‐known resource with a variety of uses. The synopsis of knowledge provided brings together previously available data but also identifies directions for future investigation, promoting more research into the complex properties of garden cress seeds, such as their potential synergies in different industrial processes and their particular therapeutic uses in health and wellness. Garden cress seeds are a viable option for industrial and nutritional uses in a world where there is an increasing need for sustainable and health‐promoting resources. This review sets the stage for continued research, innovation, and practical applications, inviting scientists, industries, and health professionals to collaborate in unlocking the full potential of garden cress seeds for the benefit of both human health and sustainable industrial practices.

## AUTHOR CONTRIBUTIONS

**Tabussam Tufail:** Supervision (equal); writing – original draft (equal). **Tehreem Khan:** Data curation (equal). **Huma Bader Ul Ain:** Writing – review and editing (equal). **Sonia Morya:** Formal analysis (equal). **Mohd Asif Shah:** Validation (equal).

## FUNDING INFORMATION

The authors received no financial support for the research, authorship, and/or publication of this article.

## CONFLICT OF INTEREST STATEMENT

All the authors declare that they have no conflict of interest.

## ETHICS STATEMENT

The authors have obtained informed consent while conducting the experiments on human participants.

## Data Availability

No data is separately available. The details of any study presented in this artile will be available on request by contacting the respective author, even though all of the pertinent data have been supplied here.

## References

[fsn34096-bib-0001] Abdel‐Aty, A. M. , Barakat, A. Z. , & Mohamed, S. A. (2023). Garden cress gum and maltodextrin as microencapsulation coats for entrapment of garden cress phenolic‐rich extract: Improved thermal stability, storage stability, antioxidant and antibacterial activities. Food Science and Biotechnology, 32(1), 47–58. 36606085 10.1007/s10068-022-01171-3 PMC9807720

[fsn34096-bib-0002] Abdel‐Aty, A. M. , Bassuiny, R. I. , Barakat, A. Z. , & Mohamed, S. A. (2019). Upgrading the phenolic content, antioxidant and antimicrobial activities of garden cress seeds using solid‐state fermentation by *Trichoderma reesei* . Journal of Applied Microbiology, 127(5), 1454–1467. 10.1111/jam.14394 31330070

[fsn34096-bib-0003] Abdel‐Aty, A. M. , Salama, W. H. , Fahmy, A. S. , & Mohamed, S. A. (2019). Impact of germination on antioxidant capacity of garden cress: New calculation for determination of total antioxidant activity. Scientia Horticulturae, 246, 155–160. 10.1016/j.scienta.2018.10.062

[fsn34096-bib-0005] Abo El‐Maati, M. F. , Labib, S. M. , Al‐Gaby, A. , & Ramadan, M. F. (2016). Antioxidant and antibacterial properties of different extracts of garden cress (*Lepidium sativum* L.). Zagazig Journal of Agricultural Research, 43(5), 1685–1697. 10.21608/zjar.2016.98127

[fsn34096-bib-0006] Abuelgasim, A. I. , Ali, M. I. , & Hassan, A. (2015). Antimicrobial activities of extracts for some of medicinal plants. International Journal of Advanced and Applied Sciences, 2(2), 1–5. 10.1016/j.sjbs.2020.08.015

[fsn34096-bib-0007] Agarwal, N. , & Sharma, S. (2013). Appraisal of garden cress (*Lepidium sativum* L.) and product development as an all pervasive and nutrition worthy food stuff. Annals of Food Science and Technology, 14(1), 2013.

[fsn34096-bib-0008] Ahmad, A. , Nabi, R. , Mishra, A. , & Ahmad, I. Z. (2021). A panoramic review on *Lepidium sativum* L. bioactives as prospective therapeutics. Drug Research, 71(05), 233–242. 10.1055/a-1334-4101 33378774

[fsn34096-bib-0009] Ahmad, R. , Mujeeb, M. , Anwar, F. , Husain, A. , Ahmad, A. , & Sharma, S. (2015). Pharmacognostical and phytochemical analysis of *Lepidium sativum* L. seeds. International Current the Pharmaceutical Journal, 4(10), 442–446. 10.3329/icpj.v4i10.24913

[fsn34096-bib-0010] Ahsan, S. K. , Tariq, M. , Ageel, M. , Alyahya, M. A. , & Shah, A. H. (1989). Studies on some herbal drugs used in fracture healing. International Journal of Crude Drug Research, 27(4), 235–239. 10.3109/13880208909116909

[fsn34096-bib-0011] Ali, R. F. M. (2013). Preparation and characterization of protein isolate and biodiesel from garden cress seed. European Journal of Chemistry, 4(2), 85–91. 10.5155/eurjchem.4.2.85-91.710

[fsn34096-bib-0012] Al‐Saad, O. A. , & Al‐Saadi, S. A. M. (2021). Chemical composition and antioxidants of *Lepidium sativum* and *L. aucheri* . University of Thi‐Qar Journal of Science, 8(1), 39–47. 10.32792/utq/utjsci/v8/1/7

[fsn34096-bib-0013] Alsadee, A. , & Agbashee, S. (2021). Hepato‐nephroprotective role of *Lepidium sativum* against oxidative stress induced by dexamethasone in rats. Indian Journal of Forensic Medicine and Toxicology, 15(1), 2643–2653. 10.37506/ijfmt.v15i1.13797

[fsn34096-bib-0014] Al‐Snafi, A. E. (2019). Chemical constituents and pharmacological effects of *Lepidium sativum* . International Journal of Current Pharmaceutical Research, 11(6), 1–10. 10.22159/ijcpr.2019v11i6.36338

[fsn34096-bib-0015] Angel, M. , & Devi, K. V. (2014). Effect of garden cress seeds incorporated health mix among selected anaemic adolescent girls (12–15 years) in Dindigul district, Tamil Nadu, India. International Journal of Scientific Research, 3, 64–66.

[fsn34096-bib-0016] Angel, M. , & Devi, K. V. (2015). Therapeutic impact of garden cress seeds incorporated ladoo among the selected anaemic adolescent girls (12–15 years). Journal of Drug Discovery, Development and Delivery, 3, 18–22.

[fsn34096-bib-0017] Attia, E. S. , Amer, A. H. , & Hasanein, M. A. (2019). The hypoglycemic and antioxidant activities of garden cress (*Lepidium sativum* L.) seed on alloxan‐induced diabetic male rats. Natural Product Research, 33(6), 901–905. 10.1080/14786419.2017.1413564 29237302

[fsn34096-bib-0018] Azene, M. , Habte, K. , & Tkuwab, H. (2022). Nutritional, health benefits and toxicity of underutilized garden cress seeds and its functional food products: A review. Food Production, Processing and Nutrition, 4(1), 1–13.

[fsn34096-bib-0019] Azimkhanova, B. B. , Ustenova, G. O. , Sharipov, K. O. , Rakhimov, K. D. , Sayakova, G. M. , Jumagaziyeva, A. B. , Flisyuk, E. V. , & Gemejiyeva, N. G. (2021). Chemical composition and antimicrobial activity of subcritical CO_2_ extract of *Lepidium latifolium* L. (Brassicaceae). International Journal of Biomaterials, 1–11. 10.1155/2021/4389967 PMC8360737 34394355

[fsn34096-bib-0020] Bathish, Y. , El Kilani, N. , Dawaba, A. , & Farid, Z. (2021). Effect of *Lepidium sativum* gel as an adjunct to non‐surgical treatment in Management of Periodontitis Patients Stage (II, III) and grade (A, B). Al‐Azhar Dental Journal for Girls, 8(2‐C), 285–292. 10.21608/ADJG.2021.48100.1315

[fsn34096-bib-0021] Besufekad, Y. , Beri, S. , Adugnaw, T. , & Beyene, K. (2018). Antibacterial activity of Ethiopian *Lepidium sativum* L. against pathogenic bacteria. Journal of Medicinal Plant Research, 12(6), 64–68. 10.5897/JMPR

[fsn34096-bib-0022] Bhardwaj, S. , Kapoor, B. , Devi, Y. K. , & Kapoor, D. (2020). Different seeds in food industry: Health benefits and Industrialapplications. Plant Arch, 20(2), 8486–8490.

[fsn34096-bib-0023] Bin Abdullah Juma, A. B. H. (2007). The effects of *Lepidium sativum* seeds on fracture‐induced healing in rabbits. Medscape General Medicine, 9(2), 23. PMC1994840 17955079

[fsn34096-bib-0024] Bin Abdullah Juma, A. B. H. , & Martin, C. R. (2011). Garden cress (*Lepidium sativum*) seeds in fracture‐induced healing. In Nuts and seeds in health and disease prevention (pp. 513–520). Academic Press. 10.1016/B978-0-12-375688-6.10061-1

[fsn34096-bib-0025] Chopra, R. N. , Nayar, S. L. , & Chopra, I. C. (1986). Glossary of Indian medicinal plants (including the supplement). Council of Scientific and Industrial Research.

[fsn34096-bib-0026] Conforti, F. , Ioele, G. , Statti, G. A. , Marrelli, M. , Ragno, G. , & Menichini, F. (2008). Antiproliferative activity against human tumor cell lines and toxicity test on Mediterranean dietary plants. Food and Chemical Toxicology, 46(10), 3325–3332. 10.1016/j.fct.2008.08.004 18768152

[fsn34096-bib-0027] Diwakar, B. T. , Dutta, P. K. , Lokesh, B. R. , & Naidu, K. A. (2010). Physicochemical properties of garden cress (*Lepidium sativum* L.) seed oil. Journal of the American Oil Chemists' Society, 87, 539–548. 10.1007/s11746-009-1523-z

[fsn34096-bib-0028] Dixit, V., Jr. , Kumar, I. , Palandurkar, K. , Giri, R. , & Giri, K. (2020). *Lepidium sativum*: Bone healer in traditional medicine, an experimental validation study in rats. Journal of Family Medicine and Primary Care, 9(2), 812–818. 10.4103/jfmpc.jfmpc_761_19 PMC7113932 32318426

[fsn34096-bib-0029] Doke, S. , & Guha, M. (2014). Garden cress (*Lepidium sativum* L.) seed‐an important medicinal source: A review. Cellulose, 9(0.03), 69–80.

[fsn34096-bib-0030] Eddouks, M. , & Maghrani, M. (2008). Effect of *Lepidium sativum* L. on renal glucose reabsorption and urinary TGF‐β1 levels in diabetic rats. Phytotherapy Research, 22(1), 1–5. 10.1002/ptr.2101 18064603

[fsn34096-bib-0031] Eddouks, M. , Maghrani, M. , Lemhadri, A. , Ouahidi, M. L. , & Jouad, H. (2002). Ethnopharmacological survey of medicinal plants used for the treatment of diabetes mellitus, hypertension and cardiac diseases in the south‐east region of Morocco (Tafilalet). Journal of Ethnopharmacology, 82(2–3), 97–103. 10.1016/S0378-8741(02)00164-2 12241983

[fsn34096-bib-0032] Eyres, L. , & Eyres, M. (2014). Flaxseed (linseed) fibre‐nutritional and culinary uses‐a review. Food New Zealand, 14(2), 26–28. 10.3316/informit.370172423621267

[fsn34096-bib-0033] Falana, H. , Nofal, W. , & Nakhleh, H. (2014). A review article Lepidium sativum (garden cress) (pp. 1–8). Pharm‐D Program, College of Nursing, Pharmacy and Health Professions.

[fsn34096-bib-0034] Ghamari, M. A. , Amiri, S. , Rezazadeh‐Bari, M. , & Rezazad‐Bari, L. (2021). Physical, mechanical, and antimicrobial properties of active edible film based on milk proteins incorporated with *Nigella sativa* essential oil. Polymer Bulletin, 1‐21, 1097–1117. 10.1007/s00289-021-03550-y

[fsn34096-bib-0035] Gokavi, S. S. , Malleshi, N. G. , & Guo, M. (2004). Chemical composition of garden cress (*Lepidium sativum*) seeds and its fractions and use of bran as a functional ingredient. Plant Foods for Human Nutrition, 59, 105–111. 10.1007/s11130-004-4308-4 15678716

[fsn34096-bib-0036] Gopalan, C. , Rama Sastri, B. V. , & Balasubramanian, S. C. (1971). Nutritive value of Indian foods. 14045465

[fsn34096-bib-0037] Gupta, N. , & Morya, S. (2022). Bioactive and pharmacological characterization of Chenopodium quinoa, Sorghum bicolor and Linum usitassimum: A review. Journal of Applied and Natural Science, 14(3), 1067–1084. 10.31018/jans.v14i3.3796

[fsn34096-bib-0038] Hacıseferoğulları, H. , Özcan, M. , Demir, F. , & Çalışır, S. (2005). Some nutritional and technological properties of garlic (*Allium sativum* L.). Journal of Food Engineering, 68(4), 463–469. 10.1016/j.jfoodeng.2004.06.024

[fsn34096-bib-0039] Hazafa, A. , Iqbal, M. O. , Javaid, U. , Tareen, M. B. K. , Amna, D. , Ramzan, A. , Piracha, S. , & Naeem, M. (2022). Inhibitory effect of polyphenols (phenolic acids, lignans, and stilbenes) on cancer by regulating signal transduction pathways: A review. Clinical and Translational Oncology, 1‐14, 432–445. 10.1007/s12094-021-02709-3 34609675

[fsn34096-bib-0040] Hazafa, A. , Rehman, K. U. , Jahan, N. , & Jabeen, Z. (2020). The role of polyphenol (flavonoids) compounds in the treatment of cancer cells. Nutrition and Cancer, 72(3), 386–397. 10.1080/01635581.2019.1637006 31287738

[fsn34096-bib-0041] Imade, O. V. , Erinfolami, W. A. , Ajadi, R. A. , Abioja, M. O. , Rahman, S. A. , Smith, O. F. , & Gazal, O. S. (2018). Effects of *Lepidium sativum* supplementation on growth and gonadotropins secretion in ovariectomized, estrogen‐implanted rabbits. Asian Pacific Journal of Reproduction, 7, 155–160. 10.4103/2305-0500.237052

[fsn34096-bib-0042] Imade, O. V. , Smith, O. F. , & Gazal, O. S. (2018). Effects of dietary inclusion of *Lepidium sativum* (garden cress) seed on plasma luteinizing hormone and reproductive performance in female rabbits. Journal of African Association of Physiological Sciences, 6(1), 79–84.

[fsn34096-bib-0043] Iswariya, V. T. , Sailaja, N. , Krishna, C. V. , & Annammadevi, G. S. (2021). Natural super‐Disintegrant agents used in various Oral solid dosage forms. Journal of Drug Delivery and Therapeutics, 11(1), 110–113. 10.1016/j.ijbiomac.2017.02.036

[fsn34096-bib-0044] Jabeen, A. , Rani, S. , Ibrahim, M. , & Mohammad, A. S. (2017). A review on *Lepidium sativum* . Indo American Journal of Pharmaceutical Sciences, 4(8), 2223–2227. 10.5281/zenodo.839541

[fsn34096-bib-0045] Jain, T. , & Grover, K. (2018). A comprehensive review on the nutritional and nutraceutical aspects of garden cress (*Lepidium sativum* Linn.). Proceedings of the National Academy of Sciences, India Section B: Biological Sciences, 88, 829–836.

[fsn34096-bib-0046] Kadam, D. , Palamthodi, S. , & Lele, S. S. (2018). LC–ESI‐Q‐TOF–MS/MS profiling and antioxidant activity of phenolics from *L. sativum* seedcake. Journal of Food Science and Technology, 55, 1154–1163. 10.1007/s13197-017-3031-8 29487458 PMC5821675

[fsn34096-bib-0047] Kagathara, V. G. , Ambikar, D. B. , & Vyawahare, N. S. (2009). Hyperte SIO‐A IMAL Models Ad PHYTOMEDICI ES: A Review.

[fsn34096-bib-0048] Khan, E. A. (2018). *Lepidium sativum* effects on regulation of reproduction, hematological, and metabolic indices in Sprague‐Dawley rats. St. Cloud state University.

[fsn34096-bib-0049] Kraft, K. (2015). Some Ayurvedic herbal combinations may have potential for the treatment of patients with osteoarthritis. Focus on Alternative and Complementary Therapies, 20(2), 104–105. 10.1111/fct.12182

[fsn34096-bib-0050] Kumar, D. , Rai, D. C. , & Kumar, S. (2017). Encapsulation process optimization of iron, L‐ascorbic acid and *L. acidophilus* with sodium alginate using CCRD‐RSM. International Journal of Current Microbiology and Applied Sciences, 6(3), 1803–1813. 10.20546/ijcmas.2017.603.206

[fsn34096-bib-0051] Maghrani, M. , Zeggwagh, N. A. , Michel, J. B. , & Eddouks, M. (2005). Antihypertensive effect of *Lepidium sativum* L. in spontaneously hypertensive rats. Journal of Ethnopharmacology, 100(1–2), 193–197. 10.1016/j.jep.2005.02.024 15955648

[fsn34096-bib-0052] Mahassni, S. H. , & Al‐Reemi, R. M. (2013). Cytotoxic effect of an aqueous extract of Lepidium sativum L. seeds on human breast cancer cells. NISCAIR‐CSIR. 10.1016/j.sjbs.2012.12.002 PMC3730895 23961228

[fsn34096-bib-0053] Malar, J. , Chairman, K. , Singh, A. R. , Vanmathi, J. S. , Balasubramanian, A. , & Vasanthi, K. (2014). Antioxidative activity of different parts of the plant *Lepidium sativum* Linn. Biotechnology Reports, 3, 95–98. 10.1016/j.btre.2014.05.006 28435800 PMC5374141

[fsn34096-bib-0054] Mali, R. G. , Mahajan, S. G. , & Mehta, A. A. (2007). *Lepidium sativum* (garden cress): A review of contemporary literature and medicinal properties. Advances in Traditional Medicine, 7(4), 331–335. 10.3742/OPEM.2007.7.4.331

[fsn34096-bib-0055] Mohamed, E. F. , & Hussein, A. M. (2017). Cress seed (*Lepidium sativum*) role in the healthy processed spread cheese and its anti‐diabetic activity. International Journal of Environment, Agriculture and Biotechnology, 2(4), 2108–2120. 10.22161/ijeab/2.4.70

[fsn34096-bib-0056] Moreira, N. F. , Sampaio, M. J. , Ribeiro, A. R. , Silva, C. G. , Faria, J. L. , & Silva, A. M. (2019). Metal‐free g‐C3N4 photocatalysis of organic micropollutants in urban wastewater under visible light. Applied Catalysis B: Environmental, 248, 184–192. 10.1016/j.apcatb.2019.02.001

[fsn34096-bib-0057] Morya, S. , Menaa, F. , Jiménez‐López, C. , Lourenço‐Lopes, C. , BinMowyna, M. N. , & Alqahtani, A. (2022). Nutraceutical and pharmaceutical behavior of bioactive compounds of miracle oilseeds: An overview. Food, 11(13), 1824. 10.3390/foods11131824 PMC9265468 35804639

[fsn34096-bib-0058] Nerkar, P. P. , & Gattani, S. G. (2012). Cress seed mucilage based buccal mucoadhesive gel of venlafaxine: In vivo, in vitro evaluation. Journal of Materials Science: Materials in Medicine, 23, 771–779. 10.1007/s10856-011-4529-7 22203515

[fsn34096-bib-0059] Oe, S. , & Gmelin, R. (1952). Purification of glycoside from Lepidum sativum by chromatography on a cellulose powder column. Arzneimittel‐Forschung, 2(12), 568–569. 13031957

[fsn34096-bib-0060] Paranjape, A. N. , & Mehta, A. A. (2006). A study on clinical efficacy of *Lepidium sativum* seeds in treatment of bronchial asthma. Iranian Journal of Pharmacology & Therapeutics, 5, 55–59.

[fsn34096-bib-0061] Patel, U. , Kulkarni, M. , Undale, V. , & Bhosale, A. (2009). Evaluation of diuretic activity of aqueous and methanol extracts of *Lepidium sativum* garden cress (Cruciferae) in rats. Tropical Journal of Pharmaceutical Research, 8(3), 215–219. 10.4314/tjpr.v8i3.44536

[fsn34096-bib-0063] Prajapati, M. R. , & Dave, P. H. (2018). Therapeutic and nutritional importance of garden cress seed. Journal of Pharmacognosy and Phytochemistry, 7(5), 140–143.

[fsn34096-bib-0064] Prajapati, V. D. , Maheriya, P. M. , Jani, G. K. , Patil, P. D. , & Patel, B. N. (2014). *Lepidium sativum* Linn.: A current addition to the family of mucilage and its applications. International Journal of Biological Macromolecules, 65, 72–80. 10.1016/j.ijbiomac.2014.01.008 24418343

[fsn34096-bib-0065] Punia, S. , & Dhull, S. B. (2019). Chia seed (*Salvia hispanica* L.) mucilage (a heteropolysaccharide): Functional, thermal, rheological behaviour and its utilization. International Journal of Biological Macromolecules, 140, 1084–1090. 10.1016/j.ijbiomac.2019.08.205 31465801

[fsn34096-bib-0066] Rahman, M. A. , Mossa, J. S. , Al‐Said, M. S. , & Al‐Yahya, M. A. (2004). Medicinal plant diversity in the flora of Saudi Arabia 1: A report on seven plant families. Fitoterapia, 75(2), 149–161. 10.1016/j.fitote.2003.12.012 15030919

[fsn34096-bib-0067] Ramadan, M. F. , & Oraby, H. F. (2020). *Lepidium sativum* seeds: Therapeutic significance and health‐promoting potential. In Nuts and seeds in health and disease prevention (pp. 273–289). Academic Press. 10.1016/B978-0-12-818553-7.00020-6

[fsn34096-bib-0068] Rani, D. , & Ahuja, M. (2017). Carboxymethylation of *Lepidium sativum* polyuronide, its characterization and evaluation as a nanometric carrier. International Journal of Biological Macromolecules, 99, 233–240. 10.1016/j.ijbiomac.2017.02.036 28238907

[fsn34096-bib-0069] Razmkhah, S. , Razavi, S. M. A. , Mohammadifar, M. A. , Koocheki, A. , & Ale, M. T. (2016). Stepwise extraction of *Lepidium sativum* seed gum: Physicochemical characterization and functional properties. International Journal of Biological Macromolecules, 88, 553–564. 10.1016/j.ijbiomac.2016.04.024 27083846

[fsn34096-bib-0070] Rehman, N. U. , Khan, A. U. , Alkharfy, K. M. , & Gilani, A. H. (2012). Pharmacological basis for the medicinal use of *Lepidium sativum* in airways disorders. Evidence‐based Complementary and Alternative Medicine, 2012, 1–8. 10.1155/2012/596524 PMC3265128 22291849

[fsn34096-bib-0072] Saju, F. , & Sivaraman, C. M. (2021). Scope of herbal mucilage in pharmaceutical formulations. A review. Herba Polonica, 67(1), 46–57. 10.2478/hepo-2021-0001

[fsn34096-bib-0073] Salehi, F. (2019). Improvement of gluten‐free bread and cake properties using natural hydrocolloids: A review. Food Science & Nutrition, 7(11), 3391–3402. 10.1002/fsn3.1245 31762992 PMC6848842

[fsn34096-bib-0074] Sat, I. G. , Yildirim, E. , Turan, M. , & Demirbas, M. (2013). Antioxidant and nutritional characteristics of garden cress (*Lepidium sativum*). Acta Scientiarum Polonorum. Hortorum Cultus, 12(4), 173–179.

[fsn34096-bib-0075] Schmitz, G. , & Ecker, J. (2008). The opposing effects of n− 3 and n− 6 fatty acids. Progress in Lipid Research, 47(2), 147–155. 10.1016/j.plipres.2007.12.004 18198131

[fsn34096-bib-0076] Sedeeq, S. H. , Al‐Layla, N. M. , & Fadhil, A. B. (2021). Biodiesel production from a non‐edible oil, *Lipidium sativum* L. seed oil by optimized alcoholysis reaction. Egyptian Journal of Chemistry, 64(10), 6075–6087. 10.21608/ejchem.2021.79680.3915

[fsn34096-bib-0077] Shawle, K. , Urge, M. , & Animut, G. (2016). Effect of different levels of *Lepidium sativum* L. on growth performance, carcass characteristics, hematology and serum biochemical parameters of broilers. Springerplus, 5(1), 1–15. 10.1186/s40064-016-3118-0 27652017 PMC5005250

[fsn34096-bib-0078] Singh, C. S. , Paswan, V. K. , & Naik, B. (2015). Exploring potential of fortification by garden cress (*Lepidium sativum* L.) seeds for development of functional foods—A review. Indian Journal of Natural Products and Resources (IJNPR), 167–175. 10.56042/ijnpr.v6i3.6267

[fsn34096-bib-0079] Singh, N. , David, J. , Thompkinson, D. K. , Seelam, B. S. , Rajput, H. , & Morya, S. (2018). Effect of roasting on functional and phytochemical constituents of finger millet (*Eleusine coracana* L.). The Pharma Innovation Journal, 7(4), 414–418.

[fsn34096-bib-0080] Strasser, H. , Abendstein, D. , Stuppner, H. , & Butt, T. M. (2000). Monitoring the distribution of secondary metabolites produced by the entomogenous fungus *Beauveria brongniartii* with particular reference to oosporein. Mycological Research, 104(10), 1227–1233. 10.1017/S0953756200002963

[fsn34096-bib-0081] Taghipour, A. , Ghaffarifar, F. , Horton, J. , Dalimi, A. , & Sharifi, Z. (2021). Silybum marianum ethanolic extract: In vitro effects on protoscolices of *Echinococcus granulosus* G1 strain with emphasis on other Iranian medicinal plants. Tropical Medicine and Health, 49, 1–14. 10.1186/s41182-021-00363-7 34496975 PMC8424884

[fsn34096-bib-0082] Umesha, S. S. , & Naidu, K. A. (2012). Vegetable oil blends with α‐linolenic acid rich garden cress oil modulate lipid metabolism in experimental rats. Food Chemistry, 135(4), 2845–2851. 10.1016/j.foodchem.2012.05.118 22980881

[fsn34096-bib-0083] Umesha, S. S. , & Naidu, K. A. (2015). Antioxidants and antioxidant enzymes status of rats fed on n‐3 PUFA rich garden cress (*Lepidium sativum* L) seed oil and its blended oils. Journal of Food Science and Technology, 52, 1993–2002. 10.1007/s13197-013-1196-3 25829579 PMC4375168

[fsn34096-bib-0084] Uphof, J. C. T. (1959). Dictionary of economic plants. Dictionary of Economic Plants.

[fsn34096-bib-0085] Valdés Fjad, S. (2006). Vitamina C. Actas Dermo‐Sifiliográficas, 97(9), 557–568. 17173758 10.1016/s0001-7310(06)73466-4

[fsn34096-bib-0086] Verma, T. , Sinha, M. , Bansal, N. , Yadav, S. R. , Shah, K. , & Chauhan, N. S. (2021). Plants used as antihypertensive. Natural Products and Bioprospecting, 11, 155–184. 10.34172/PS.2021.68 33174095 PMC7981375

[fsn34096-bib-0087] Wadhwa, S. , Panwar, M. S. , Agrawal, A. , Saini, N. , & Patidar, L. P. L. (2012). A review on pharmacognostical study of *Lepidium sativum Lepidium sativum* . Advance Research in Pharmaceuticals and Biologicals, 2(4), 316323.

[fsn34096-bib-0088] Xiao, J. , Muzashvili, T. S. , & Georgiev, M. I. (2014). Advances in the biotechnological glycosylation of valuable flavonoids. Biotechnology Advances, 32(6), 1145–1156. 10.1016/j.biotechadv.2014.04.006 24780153

[fsn34096-bib-0089] Youssef, G. M. , El‐Ghamery, H. E. , & El‐Sawy, H. A. E. (2014). Study the physico‐chemical properties and antihyperlipidemic activities of garden cress seed oil. Journal of American Science, 1, 324–330.

[fsn34096-bib-0090] Zanvar, V. S. , & Rohini, D. (2007). Biofortification of biscuits with garden cress seeds for prevention of anaemia. Asian Journal of Home Science, 2(1/2), 1–5.

[fsn34096-bib-0091] Zeilab Sendijani, R. , Abedian Kenari, A. , Smiley, A. H. , & Esmaeili, N. (2020). The effect of extract from dill *Anethum graveolens* on the growth performance, body composition, immune system, and antioxidant system of rainbow trout. North American Journal of Aquaculture, 82(2), 119–131. 10.1002/naaq.10123

[fsn34096-bib-0092] Zia‐Ul‐Haq, M. , Ahmad, S. , Calani, L. , Mazzeo, T. , Rio, D. D. , Pellegrini, N. , & Feo, V. D. (2012). Compositional study and antioxidant potential of Ipomoea hederacea Jacq. and *Lepidium sativum* L. seeds. Molecules, 17(9), 10306–10321. 10.3390/molecules170910306 22932212 PMC6268377

[fsn34096-bib-0093] Zia‐Ul‐Haq, M. , Ćavar, S. , Qayum, M. , Imran, I. , & de Feo, V. (2011). Compositional studies: Antioxidant and antidiabetic activities of *Capparis decidua* (Forsk.) Edgew. International Journal of Molecular Sciences, 12(12), 8846–8861. 10.3390/ijms1212884 22272107 PMC3257104

